# What’s in it for me? A process evaluation of the implementation of a mobile phone-supported intervention after stroke in Uganda

**DOI:** 10.1186/s12889-019-6849-3

**Published:** 2019-05-14

**Authors:** Minna Teriö, Gunilla Eriksson, Julius T. Kamwesiga, Susanne Guidetti

**Affiliations:** 1 Department of Rehabilitation medicine, Danderyds University Hospital, Danderyd, Sweden; 2 Division of Occupational Therapy, Department of Neurobiology Care Sciences and Society, Karolinska Institutet, Box 23 200, S-141 83 Huddinge, Stockholm Sweden; 3 Department of Neuroscience, Rehabilitation medicine, Uppsala University, Uppsala, Sweden; 4 Uganda Allied Health Examinations Board, Kampala, Uganda

**Keywords:** Stroke, Africa, ICT, Low-income, Occupational therapy, Process evaluation, SMS, Tele-rehabilitation, Tele medicine

## Abstract

**Background:**

The prevalence of stroke in Uganda is increasing. In stroke rehabilitation, information and communication technology has been shown to have potential in improving service delivery in high-income countries but there is limited knowledge of its use and impact in low-income countries.

The aim of the study was to evaluate the implementation process of a mobile phone-supported family-centred rehabilitation intervention and to gain knowledge on the mechanisms of impact as well as the contextual factors that might have affected the implementation process and its outcome.

**Method:**

This was a single-case study design using the integrated Promoting Action on Research Implementation in Health Services framework and the Medical Research Council guidance as frameworks**.** Quantitative process data was derived from 14 log books used by occupational therapists during the implementation. Qualitative semi-structured interviews were conducted with 12 implementers in different professions, 12 months into the implementation, in order to obtain the primary data. Secondary data was derived from six semi-structured interviews conducted directly after pre-intervention workshops and 6 months later. The framework method was used in the data analysis.

**Results:**

In 11 out of 14 cases, the clients were compliant with the intervention. Yet, challenges such as technical problems were reported. The target of conducting 16 phone calls for each client was achieved to 74%. Eight categories emerged from the qualitative analysis of the interviews including: 1) perceptions on facilitation, 2) using scientific and experience-based knowledge, 3) tailoring the intervention, 4) supportive working culture, 5) barriers to the service delivery, 6) implementers’ interaction with the intervention, 7) perceptions on motivations and values, and 8) improving the model and enabling sustainability. Mechanisms contributing to the implementation of the intervention included engaged facilitators and motivated participants. Challenges in the client recruitment and poor information dissemination were some of the mechanisms impeding the implementation.

**Conclusions:**

The intervention was partially delivered in accordance with the logic model for the project, where the implementation process was influenced by several barriers in the context such as technical setbacks. However, there were also several mediators in the process driving the project forward, including strong facilitation and motivated participants.

**Electronic supplementary material:**

The online version of this article (10.1186/s12889-019-6849-3) contains supplementary material, which is available to authorized users.

## Background

Estimations by the World Health Organization indicate that stroke is currently ranked number five among the leading causes of mortality in Uganda [1]. Despite the increased disease burden [2], the country lacks national operational policies or programmes for prevention or treatment of non-communicable diseases (NCDs) including stroke [3]. In Uganda, 72% of households live within a radius of five kilometres of a healthcare facility. However, limited utilization of the facilities has been reported, related to factors such as lack of means of transport, poor infrastructure, human resource shortages and lack of incentives for the staff. The healthcare service delivery in Uganda is regulated by the government and supported by private sectors [4]. The Ministry of health is responsible for the planning and allocation of finances to individual health units. The private wings of public hospitals and healthcare at private health facilities is financed by out of pocket payments from the patients. Healthcare is delivered at specialized tertiary level by the National Referral Hospitals, such as Mulago hospital in the capital Kampala, to which people with stroke are admitted. At secondary level care is provided by Regional Referral Hospitals with narrower specialization. Primary care is provided by healthcare units in the communities with variations in the service capacity. The private healthcare sector is responsible for about 50% of healthcare service delivery [5].

### Mobile technology in healthcare service delivery

The UN has introduced the Sustainable Development Goals (SDGs) – a 17-point plan to end poverty, combat climate change and fight injustice and inequality by 2030 [6]. The mobile connectivity will play a central role in the implementation of the SDGs in Sub-Saharan Africa, facilitated by the fact that the digitalisation is growing fast. There are currently over 1000 mobile health services in low-income countries providing health content and diagnostics services. [7] One of the SDGs goals focuses on good healthy lives and promoting well-being for all. Bearing this in mind, the use of mobile phones can increase the quality, reduce the costs and extend the reach of healthcare to benefit millions. In the East African countries incorporation of mobile phones have been fast [8]. In sub-Saharan Africa in 2017, there were 444 million people subscribing to mobile phones [7]. A mobile broadband network now covers most urban areas, and thus the infrastructure-related exclusion is greatest in rural areas. However, the expansion of conventional network infrastructure is more challenging. Typically, in sub-Saharan Africa, 20% of the population spreads over 70% of the territory. In many cases, a difficult terrain such as mountains and forests might be a hinder and challenge for the expansion of the infrastructure. Further, income from rural areas is only about one tenth, unlike in urban areas. This is because most rural areas have low purchasing power, returns, taxes and energy accounts for up to 60% of the cost of mobile broadband. [7] In Uganda the use of mobile phones has spread progressively throughout the country since 1995 [7–9] and 2017 mobile phone subscriptions was 58 per 100 habitants [10]. This indicates that there is potential for successful use of mobile phones to increase the accessibility of healthcare services [8], especially due to an increasing societal demand for ICT use in people’s daily activities [11].

The potential of using Information and Communication Technology (ICT), including mobile phone solutions among stroke survivors, is supported by previous studies [12–15], but more research is needed. Some studies conducted in high-income settings suggest that ICT can be used as a tool for communication between healthcare professionals and clients in home care, in order to promote and improve the performance of activities in daily living (ADL), or it can be used as complementary service [13, 14, 16, 17]. The term telerehabilitation is also used and refers to any ICT supported rehabilitation service provided for people in their homes or other environments due to long distances from healthcare facilities [18]. Despite several challenges in healthcare and rehabilitation in Uganda, new ways of providing services might create a solution to tackle some of the problems.

Additionally, to ensure the efficiency and sustainability of ICT-based services, the services need to be carefully integrated into the local context [8, 19]. Until today, no research is available on the use of telerehabilitation in stroke care in low-income settings such as Uganda.

This study is a process evaluation [20] of the implementation of a mobile phone-supported family-centred rehabilitation intervention F@ce*™*, in Uganda and focuses on the processes at the implementation level of the intervention. Moore et al. [21] suggest a process evaluation as an essential part when designing and testing complex interventions, and that this plays an essential role in gaining an understanding of the functioning of the intervention. Further, process evaluations can contribute to identifying mediators supporting the implementation as well as mechanisms hindering the process [21]. A complex intervention is usually described as an intervention containing several interacting components. The delivery of the interventions may require different skills and behaviours from the healthcare professionals involved. Complex interventions may also have an impact on several different levels in communities or organizations [20]. In the literature on rehabilitation in Uganda, no studies with a focus on process evaluations were found.

### The intervention and the implementation process

The F@ce™ intervention was implemented and studied in this study, where F stands for (Face-to-face between the OT and the client), @ for Assessment, C for Collaboration and E for Evaluation. The intervention was modelled on a Swedish client-centred ADL intervention [22] during a series of collaborative workshops to be used in the Ugandan context (see Additional file 1). A logic model for the implementation process was outlined, describing the needed and available resources, planned activities, outputs for the process, the potential short and-long term outcomes and impacts of the intervention (see Additional file 2).

The first step in the implementation included identifying and recruiting clients on different healthcare facilities. After informed consent they were allocated to intervention group (IG) or control group (CG). The local facilitator and one occupational therapist (OT) thereafter carried out a home visit to the client. The Canadian Occupational Performance Measure (COPM) [23] was used to assist clients to set three targets regarding activities they wanted and needed to do. The client in IG were informed about and supposed to use a problem-solving strategy as a basic structure for planning and practicing the chosen activities. The client and the family members also received written information about the agreed targets and the strategies for reaching them.

During the eight weeks of intervention, the three set activity targets were to be delivered to the client every morning and evening by short message service (SMS). The morning SMS was a reminder to perform the activities during the day. In the evening the client was supposed to respond in three separate SMSs by scoring the performance of the activity between 0 (has not performed the activity) and 5 (did the activity well). If the clients had scored 0 or had not responded to the SMS, a red flag (a message that informed of a non-performed activity) was sent to the OT who the following morning should call the client to solve the problem. Additionally, the clients were to receive calls from OT twice a week as a follow up.

The CG clients underwent similar assessments as the IG but did not receive reminder SMS or phone calls. The local facilitator also provided hand therapy balls and measured blood pressure of the clients in both groups.

This study aimed to evaluate the implementation process of a mobile phone-supported family-centred rehabilitation intervention and to gain knowledge of the mechanisms of impact, as well as the contextual factors that might have affected the implementation process and its outcome.

## Methods

**Fig. 1:**
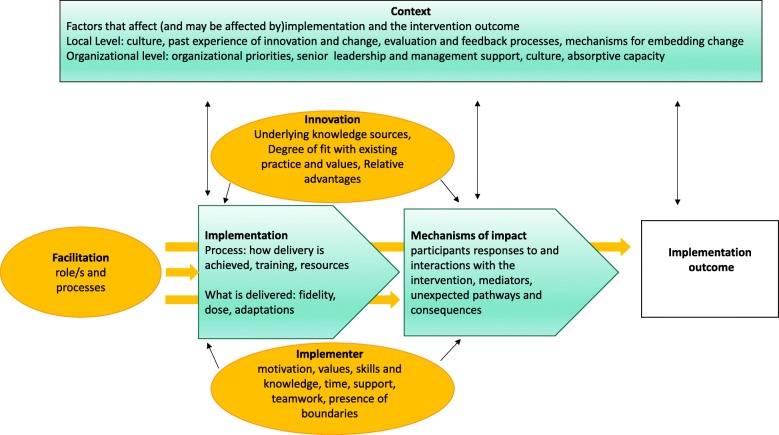
The key components in a process evaluation. Key components of a process evaluation and constructs of i- PARIHS in a combined framework. Adapted from: Moore et al. [21] and Harvey G, Kitson A [24]

Moore et al. suggests that drawing upon a suitable theoretical framework can be useful for the evaluators when conducting process evaluations [21]. The current study used two main frameworks. The Medical Research Council (MRC) guidance [20] was used to identify the key processes of the implementation process and the Promoting Action on Research Implementation in Health Services (i-PARIHS) [24] to define areas of investigation related to the key components in a process evaluation (See Fig. 1). The i-PARIHS framework describes four dimensions including 1) innovation, 2) recipients, 3) context and 4) facilitation which could influence the implementation outcomes where the facilitation is considered as a driving element [24]. In this study the original i- PARIHS concept of “recipients” is re-named “implementers” to be more in line with the MRC guidance [20], since the word recipient may emphasize a rather passive role for the persons involved in the implementation process [24]. This term is also suitable since this study incorporates only the implementers’ perspectives. The perspectives of other participants, such as patients and caregivers will be presented elsewhere.

### Study design

The study used a single-case study design using mixed methods including semi-structured interviews and quantitative process data [25, 26].

### Study setting

The process evaluation was carried out by two evaluators within the context of a research project in Uganda. The purpose of the main research project was to evaluate the feasibility of a model for mobile phone-supported and family-centred rehabilitation intervention to increase functioning in daily activities for persons living with the consequences of stroke, as well as participation in everyday life for persons with stroke and their family members [27].

The planning of the research project was initiated in September 2015 followed by workshops for modelling the intervention and a training workshop for five occupational therapists (OTs). The inclusion of clients in the intervention was ongoing during March 2016 to February 2017.

The intervention was implemented in urban Kampala and within 40 km of the city. Included were 30 clients who had had a stroke, of these, 15 in the intervention group and 15 in the control group. The clients were recruited from two healthcare facilities and through Facebook, but the services were mainly delivered in the clients’ home environment and at a distance by using a mobile phone. The outcome of the intervention is presented elsewhere. This process evaluation includes analysis of data for the time period 2016–2017 when the implementation to evaluate the feasibility of the intervention occurred.

### Participants

**Fig. 2:**
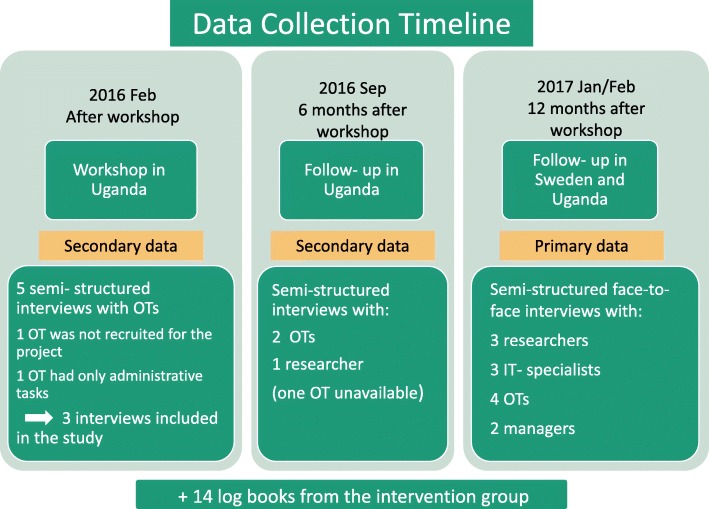
The data collection timeline. Data collection timeline and data sources

To ensure rich data and variation in the responses, the selection of study participants was done by purposive sampling [25], resulting in a total of 12 participants; four OTs, three researchers, three information technology (IT) specialists and two rehabilitation managers (See Fig. 2).

All the participants described here were considered as implementers in this study, but they played different roles. The researchers who had planned the research project were also facilitators. One researcher functioned as a local implementer/facilitator and had the main responsibility for the facilitation in Uganda. Five local occupational therapists had been recruited to deliver the intervention and had participated in a preparation of eight half-day workshops. Four of the therapists were interviewed for this study. The OTs took part in the present project outside their ordinary working time. The managers provided a platform for the client recruitment at their units.

### Data collection

Data from a mixed data set was used by two evaluators who were blinded to the trial outcome. The evaluators were an occupational therapist (the first author), and a nurse respectively (a research assistant with Ugandan origin). Both the evaluators had previous working experience from Uganda. They were not part of the implementation team, and this was considered to be an advantage, and might increase trust in the interview situations as well as reduce potential researcher- and response bias.

Before collecting the primary data, the authors familiarized themselves with the secondary data by reading the interview guides for those interviews and listening to audio recordings in order to identify areas which needed further investigation and to develop the interview guides for the primary interviews. The interview guides were then developed by a deductive approach to cover the main components from the MRC [20] and i- PARIHS) [24] frameworks to identify the key processes of the implementation. Additional questions were included to encourage the participants to reflect on a possible need for improvement in the intervention and the implementation process.

The primary data included twelve interviews which were conducted by the first author and the research assistant 12 months after the OTs training workshop in 2017. The interviews were audio-recorded and conducted in the participants’ native language; Swedish or English was used depending on the participants’ country of origin. This was possible since the evaluators were fluent in both languages.

Secondary data consisted of six semi-structured audio-recorded interviews conducted by the two main researchers in the study (the second and the fourth author). They were collected within six months of the workshop in 2016 (Fig. 2). The duration of the interviews varied from 20 to 90 min. Additionally, in order to study what was delivered, data were collected from 14 logbooks written by the OTs, one for each client in the intervention group. One logbook was not available during the data collection for unknown reasons.

### Data analysis

All the interviews were transcribed verbatim, and the framework method was used in the data analysis of the interviews [28]. After the initial line-by-line open coding of three initial interviews, the codes were discussed between the two evaluators. An analytical framework with unified codes was developed to be used in systematically indexing the subsequent interviews. The two evaluators interpreted and discussed the cases after charting the data in a framework method matrix [28] to ensure transparency and internal validity of the findings.

Analysis of the process data was derived from the handwritten log books. The data from logbooks was summarized by case and organized in a table in Microsoft Excel. By using Excel it was possible to make the data quantitative, for example by counting how many times the OTs had reported on problems or advances in the intervention.

To enhance credibility, the findings were also discussed with the co-authors during the last steps in the analysis and critical questions were continuously discussed regarding the findings. Finally, the results were presented and discussed with the experienced research group as a form of informal triangulation.

## Results

The analyses from the mixed dataset revealed several improvements and challenges in the implementation process as well as variations in the delivery of the intervention. The first part of these results illustrates the process data gathered from the log books. The second part reveals results from the analysis of the 18 qualitative interviews among the 12 participants in the study (Fig. 2).

**Fig. 3:**
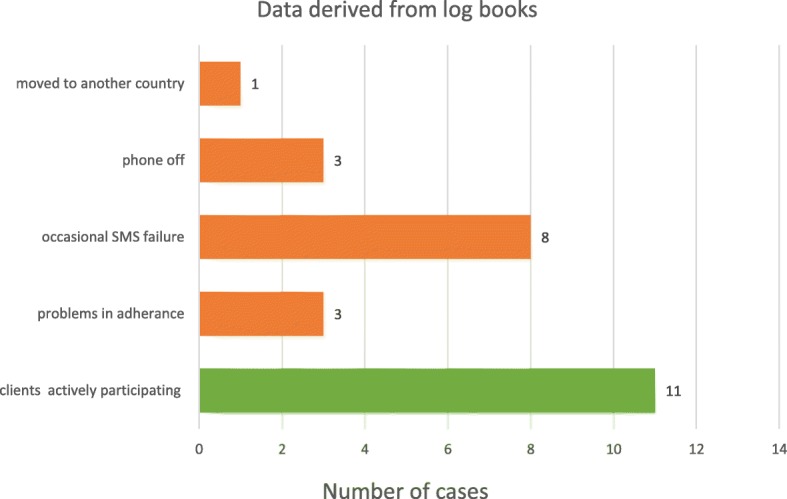
Information gathered from the logbooks written by the OTs. Eleven cases showed the clients were adherent to the intervention. Problems reported

The 14 logbooks written by the OTs reported several advances in the service delivery. For example, in 11 cases, the clients were adherent to the intervention with support from their caregiver i.e. a family member who mainly helped and lived together with the person with stroke. However, several problems were also reported, such as the clients not receiving a reminder SMS (See Fig. 3).

Table 1 shows the number of phone calls conducted by OTs for each client in the intervention group. The clients were to receive calls from OTs twice a week. The logbooks showed that the target of 16 calls per client was achieved by 74%.

**Table 1:** Number of phone calls conducted by the OTs

Client	1	2	3	4	5	6	7	8	9	10	11	12	13	14	15	Mean	Target	Reach
Number of calls	6	9	13	12	10	13	14	14	13	11	14	12	12	13	12	12	16	74%

**Fig. 4:**
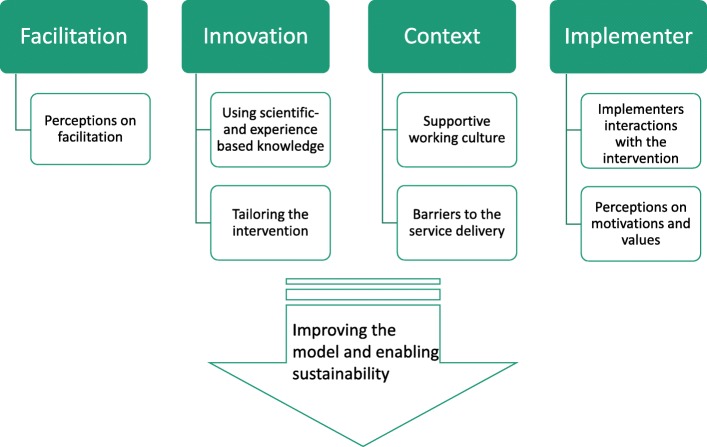
The emerging categories related to the main constructs in the combined theoretical framework. The relationships between the eight categories that were identified in the qualitative interviews

Eight categories were identified in the qualitative interviews, out of which seven categories were connected to the four constructs in the combined theoretical framework. The findings under these categories revealed mechanisms and contextual factors that may have influenced the implementation processes. An additional independent category emerged from the participants’ responses related to the improvement suggestions. Relationships between the categories are shown in the Fig. 4**.**

### Perceptions on facilitation

All participants expressed appreciation of the facilitation and the different methods used in the implementation of the intervention. All the facilitators were perceived to be helpful and fully engaged in the project. The workshop in Uganda promoted collaboration and contributed to unifying the team and the teamwork. One OT said: “*I like the bit where we have been sharing totally, the discussing at almost same equal levels*”, meaning the different OTs could share their views openly with the researchers without hierarchy and despite different educational levels. The good communication was also appreciated by the participants.

The local facilitator and the OTs communicated with each other when necessary including, for example, when problems occurred with SMS sending which therefore affected the delivery of the intervention. In Uganda, most of the communication was done by phone calls among the implementers, but in Sweden, communication was mostly by e-mail. Even so, there were challenges in the communication when, for example, mishaps occurred in the e-mail communication which caused delays in finalizing the IT-structure.

Most of the participants shared the perceptions of having sufficient resources to accomplish their tasks in the project. Yet, several participants also mentioned that they would have needed more support and information from the IT technicians, since lack of knowledge of the technical parts of the intervention made some tasks, such as handling the monitoring of SMS sending difficult to handle. Another challenge mentioned by some participants was agreeing on the incentives for the work of OTs. This issue was, however, resolved by signing up contracts for payment for service to OTs at the beginning of the implementation.

A mechanism of impact seemed to be that there was a need of facilitators both at a local level and at an overall project level, using methods for communication that were accepted in the cultural context.

### Using scientific and experience-based knowledge

Integrating scientific evidence- and experience-based knowledge related to the know-how of the local context seemed to be required in the modelling of the intervention and in the implementation process. The scientific evidence and practice were integrated by sharing material and discussing the evidence in the workshop. However, when discussing the design of the client-centred intervention, the research- and practice-based knowledge came into conflict. The OTs required an adjustment to be made in order to adapt the intervention to the local context. One of the researchers said “*It has to be family-centred, and we thought that we would interview stroke victims and relatives separately. It did not work out that way, because both stroke victims and family members speak as a unit. [...] We changed for them so that they would feel comfortable with the choice”.* Thereby, there was a need to change the method and the term “client-centred” to fit into “family-centred” to meet the Ugandan OTs preferences.

The OTs appreciated the access they had to scientific articles in the project. They believed that the new knowledge acquired made a difference in their clinical work. One participant said: *“Rehabilitation will be more successful if someone is involved in goal-setting, and that is another paper which we reviewed. So those are things, eye-openers, which we didn’t know and we are getting them from this workshop”.* Another OT thought that the new knowledge led to a change of approach to clients, and said*: “I could look at the patient not only as an object but as someone who has been affected and needs a lot of understanding”.* Furthermore, several participants expressed having sufficient knowledge to carry out the intervention, not only as a result of the new knowledge they had received but also due to their previous working experience.

One mediating mechanism of impact was to match the implementers’ earlier professional education, their working experience and scientific knowledge of the new intervention.

### Tailoring the intervention

To be able to implement the new intervention, several adjustments in the modelling of the intervention and the implementation processes were needed in order to fit the specific context. It was planned that a server should be placed in Uganda, but for practical reasons, the server was placed in Sweden instead. Thereby, Swedish phone numbers needed to be used for the communication between the clients and the OTs. The Swedish server caused unexpected challenges since the local facilitator needed to send airtime for each individual client on a daily basis for international SMS from the local teleoperators.

The clients were using their own phones since stroke could be expected to cause problems in learning to use the new technology. Technical restrictions appeared when not using smartphones since the targets formulated were required to fit in the SMS function with a limited number of letters.

During the project, the OTs and the local facilitator were provided with tablets, which were to be used in the intervention. The original function of the tablets included filming the clients in order to enable the self-assessment by watching the video. This was not implemented due to time constraints during the home visits and discomfort to the clients. One OT said:

*“You see these tabs are very good and you can take a photo, but we realized that we can’t easily use them, because when you start to film a client, uhm they become eeeh the mood really changes.*”

The tablets were also seen to be somewhat unpractical for use in the local environment due to the risk of being robbed in public places.

There were several challenges in the implementation process of the project. Being flexible and adapting the intervention to the current context is a must when implementing new interventions.

### Supportive working culture

Several participants described that the need for understanding cultural differences and having trust and flexibility was essential in establishing good collaboration. Most participants expressed satisfaction at being a part of the project and described the positive working culture. As one participant stated: “*I think we have very fantastic working relationships. I say we have not developed any bad relationship, it is because relationship it is based on what? Work”.* Some of the OTs said that the discussions within the workshop about the challenges in their clinical work were perceived as helpful.

The participants’ familiarity with each other was a common theme in the responses, and this motivated them to participate in the project as well as to give support to one another: *“So we believe in each other and trust each other. Had it been an unknown or so to say a new acquaintance that I’ve never worked with on a project, then I do not know if I would have been happy”,* as one participant expressed his thoughts about the working environment*.*

### Barriers to the service delivery

There were several barriers and mechanisms described in the implementation process that might have influenced the outcome of the research project. One of the main challenges faced was the recruitment of the clients. This was due to several reasons including high mortality and not getting access to healthcare facilities; this last challenge applied especially to the private profit organizations. Lack of engagement among local colleagues or other medical personnel provided an additional barrier to client recruitment and was reflected in: *“We tend to be focused on the now, so when I quickly look and there isn’t anything for me now then, I don’t get interested, so it’s not necessarily our own problem; I think it’s a local problem (IT-specialist)”.* He expressed disappointment at the lack of interest and that people were not paying attention to the relevance of the intervention. Another contextual barrier affecting client recruitment was the lack of knowledge of stroke symptoms, which was evident when the local facilitator received potential clients with conditions other than stroke.

Factors expressed by several participants that might have influenced the implementation process were dishonesty, non-compliance by clients and the family members as well as doubts on how beneficial the research would be. For example, the airtime sent to the clients was at times used for other purposes by the family members or by clients, which was detected when monitoring the SMS sending through the server. Furthermore, according to the local facilitator, some medical staff at the healthcare units involved had expressed doubts on how beneficial the research would be for the clients.

There were also challenges when conducting follow-ups with the clients. At times, the phones were switched off or the network failed. One OT explained: “*You would be calling the patients for almost three to four days without getting in touch*”. In some cases, the family members that were handling the phones travelled away, which also complicated the follow-ups.

According to the IT-specialists, an unexpected server breakdown, and the fact that the international SMS might have been blocked by local tele operators in Uganda were some of the hindrances in the communication between the clients and the OTs.

Several participants mentioned that the SMS function was not clearly defined when the project started. Due to the unclear SMS format, the implementers experienced challenges: *“The other complication was teaching them how the message, what information they would send to the server because we had not agreed properly in the workshop how the message would look”.* Lastly, another challenge occurred when the OTs and the local facilitator tried to teach the clients how to send the SMS; they found that the process was difficult to explain in the local language.

### Perceptions on motivations and values

The reasons and motives to participate in the research project as well as the expectation of the individual benefits varied among the participants. All participants described that they benefitted from the project in some way, and some thought they were improving their professional working methods, or that the project should raise public awareness of stroke.

Several of the participants expressed a pride in participating in the project and said that they often made reference to it. One participant said; *“So, I think this is a breath of fresh air; it is something to put your hands to so that they will make something which will help people and that’s I guess one of the most important things we have* [translated from Swedish]*”,* meaning that this aspect made the project more valuable than many other projects he worked with.

Some participants expressed having doubts about the intervention at the start, but also how they had now had greater trust in the effectiveness of the intervention. As one OT said: *“I had never seen it working, so I had doubts as well that it can work or that it may not work. So, I was on a 50–50 range, it may or it may not”.* But the doubts faded later in the project. However, several participants expressed their doubts about the sustainability of the implementation of the intervention to the Ugandan healthcare system due to a lack of financial support in the hospitals.

### Implementers’ interactions with the intervention

This category represents the participants’ general reflections on different experiences during the implementation when interacting with the clients, caregivers or other stakeholders involved in the intervention.

To work with a client-centred approach in the intervention, clients’ interests were used in planning the rehabilitation and meaningful targets were chosen together with the clients. However, the OTs had observed that the caregivers, who most often were family members, had a great impact on the rehabilitation. For example, they were often operating the SMS and phones on behalf of the clients. On the other hand, they were also affected, since they needed to give up a lot so as to support their relatives during the post-stroke period.

During the implementation of the intervention, several participants assumed additional tasks due to several unexpected events. That was also challenging when having different perceptions of the responsibilities and tasks among project stakeholders. For example, in some units, the local facilitator was expected to do more practical work with the clients than originally planned. According to the local facilitator, this might have caused some problems in the collaboration. Furthermore, the managers reported that they had not received sufficient information concerning the project or its potential impacts on their unit and stressed the need of having increased “ownership” of the project. In contrast to these challenges in the collaboration, the OTs contributed to identifying potential clients when difficulties in client recruitment became reality.

### Improving the implementation model and enabling sustainability

Several different ideas on how the intervention or the process of implementation and adjustments in the working methods could be improved in the future were suggested by the participants for better operationalization of the intervention. For example, some participants thought that the intervention could have been carried out with ordinary phones instead of tablets. One OT said: “*It can really work, it can, this is very good, ah this is a good, a very good approach to rehabilitation, you see and can really, very, it’s very cost-effective […]*, *all we need is a calling and a phone.*” It was also suggested that setting targets earlier and establishing therapeutic relationships with the clients through increased contact time and “hands-on therapy” before discharge from the hospital could increase clients’ trust and could lead to better outcomes.

There were also several suggestions on the technical improvement of the intervention, especially if expanding the project. One IT- specialist recommended, *“It is very important in the beginning when you build this kind of software that you know how many users there will be/how many people were going to take care of, and what the SMS will look like. They’re small details, but very important to think about* [translation from Swedish]”. Some participants thought that the functioning of the system could be improved by close collaboration with local tele operators and having a server in Uganda, which would also ensure lower costs for the services provided.

Some adjustments of the client recruitment procedures were also suggested, such as trying to recruit clients in primary hospitals. One manager suggested that providing better incentives to the clients could lead to increased access to clients*: “I think it requires some funds……. they want some facilitation to motivate them, as much as you tell them it’s going to help others, but they say umh, how will I benefit from it”.* The manager also said that their organization might need monetary incentives to participate in similar research projects in the future. In order to attract different stakeholders and policy makers, raising public awareness regarding stroke was further suggested. One participant addressed the need of a top-down approach if the intervention was to be integrated in the Ugandan healthcare system. In summary, to improve the implementation model and the intervention, the participants suggested adjustments to be made in the working methods and incentives. They further emphasized the need of improved information dissemination to all stakeholders so as to ensure engagement and sustainable integration of the intervention.

### To what extent was the intervention delivered as intended?

When comparing the content of the logic model from the research project with the participants’ responses in the interviews, the evaluators concluded that most of the planned activities, such as training workshops, regular meetings, client assessments and support from researchers were implemented as intended with satisfaction on the part of the implementers. Yet, as seen earlier in the results section, the target of conducting follow-up phone calls to the clients twice a week was not achieved for any of the clients. Further, it remained unclear how often the clients received or sent SMS in the intervention, since this was not technically possible to observe during the project.

## Discussion

This study has evaluated the implementation process of a mobile phone-supported family-centred rehabilitation intervention in Uganda and has gained knowledge on the intervention mechanisms of impact as well as contextual factors that might have affected the implementation process and its outcome. The results confirmed advances, but also a variety of challenges, in the implementation process. These could vary from technical problems to doubts about the research as expressed by different medical personnel, which reflected the complexity of the implementation process. However, despite the challenges, several mediators such as engaged facilitation, as well as highly motivated participants, were the driving factors in overcoming the challenges and thereby completion of the research project. The mediators and barriers and their relations to the core components of the theoretical framework (Fig. 1) are further discussed below.

### Facilitation

The facilitation was structured at different organizational levels in this intervention, being in line with the i- PARIHS model of having novice, experienced and expert facilitators involved within the project (25). The division of the tasks may have provided a vital component in the success of facilitation, by for example having different responsibilities on different levels in the implementation, thus enhancing the creation of supportive working culture for all members in the implementing team. Further, the i-PARIHS framework capitalizes on the fact that the facilitator’s ability to empower participants is vital in making the implementation successful [24]. This idea of empowering leadership was reflected in the project when facilitators organized workshops characterised by open, non-hierarchical discussions.

### Innovation

Tailoring the intervention to local preferences is one of the success factors according to the innovation construct in the i-PARIHS framework. It leads to higher degree of fit with existing practice and values, which in turn increases the acceptance of the persons involved in the intervention [24]. The facilitation method where the facilitators involved the OTs in the planning phase of the intervention resulted in necessary adaptations, such as making the intervention “family-based” instead of “client-centred”, a term often used in high-income healthcare settings [29–31]. The innovative use of different knowledge sources in terms of integrating relevant research-based knowledge into practice seemed to be one of the driving mediators, even in this implementation process.

Another component which plays an important role in the success of an implementation, is how much the implementers value the new intervention compared to their current practice [24]. The OTs in this study seemed to share the opinion of the new intervention having more advantages compared to how they normally work, which may have contributed to their acceptance.

### The context

One of the most important contextual mediators in this project was the supportive working culture. This was expressed by the participants as having a willingness to receive and give team support between members and being familiar with each other. These features are corroborated by the i-PARIHS framework, which suggests that, for successful implementation, the environmental aspects such as a favourable working culture and good leadership are vital [24].

### Implementer

According to- i-PARIHS framework, one of the influential components in an implementation process is the participants’ motivations and skills (Fig. 1). These individual factors can be considered as mediators since all the participants seemed to have clear underlying motivations for joining the project, despite the fact that those motivations could vary among the participants. However, from the responses, the evaluators concluded that a monetary incentive was the most important incentive for several of the participants. Gunberman [32] draws on several motivational theories and states that most of the people in an organization have different preferences regarding the work rewards. These preferences can vary between valuing so-called instrumental rewards such as money, and being satisfied when performing interesting tasks matching one’s own skills. Gunberman further suggests that, in healthcare management, it is essential for leaders to gain knowledge about what motivates employees when aiming to produce good performance and improvement [32].

The i- PARIHS framework suggests a sense of “ownership” as being a vital component in the success of an implementation [19]. It was speculated by the participants that managers in several private healthcare units may have chosen not to take part in the project as they perceived that they did not benefit from it. It may be that several stakeholders, including the clients, who participated in the project, did not feel ownership towards the intervention due to lack of sufficient information. Therefore, the authors suggest that increased information in future projects could help the stakeholders answer the important question “What’s in it for me?” and thereby increase their motivation to participate.

### Suggestions for improvement

Several challenges were faced largely in the operationalization of the technical parts of the intervention, which several participants also thought should be improved in the future. The importance of having well-functioning technology in telerehabilitation must not be underestimated since the technology plays a significant role in transferring information [18]*.* By involving the users in the development process of the intervention, through a user-centred design, more user-friendly solutions with fewer errors would be created [18]*.* Additionally, by clearly defining the methods for performance measurement, for example, by choosing relevant process indicators for the monitoring [33], the implementation could be improved, thus reducing lack of clarity in the monitoring and evaluation of the implementation.

The assessment procedure in the research project was seen as time-consuming and causing a burden on the clients. Previous studies have suggested that a high burden on clients may influence study enrolment and retention [34, 35]. With this in mind, in future studies, it would be beneficial to reduce the number of assessment instruments used.

## Implications of the study

Digitalisation is driving rapid technological progress and growth, generating significant benefits for consumers in both high- and low-income regions of the world. However, the rapid innovation of different technology models needs to be evaluated and implemented. Policymakers worldwide, including many in Africa, are now aware of these challenges and are working to implement reforms that enable a digital society. Therefore, there is a need for cooperation between governments, the mobile phone industry and the healthcare sector to formulate policies and programs that also include rehabilitation using ICT. Resources for these innovations are needed. In addition, an environment that enables research and evaluation of innovative programs is vital.

This study identified positive aspects and setbacks related to the intervention and its implementation. Further, it also addressed areas that require improvement; these factors would to be important if the project is to be scaled up to a full randomized controlled trial in the future. The design of this study was a qualitative single-case study with a small number of participants investigating processes in a specific context. Considering this, the generalizability of the study findings can be seen as limited. However, additional implications of this study are providing insights to processes and mechanisms leading to change when implementing complex interventions using ICT in Uganda and possibly other low-income countries. The authors suggest that several parts of the findings can be transferred to similar projects with a global perspective and that lessons can be learned from the findings. It may further provide guidance to other future implementers of ICT-based healthcare interventions. As an example, although the MRC guidance and i-PARIHS [20, 24] frameworks that were used in this study were originally developed in high-income settings, they seemed to provide appropriate guidance even for the implementation and evaluation of the intervention in a low-income country. Furthermore, the i-PARIHS framework has recently been used in a process evaluation within a quality improvement project in rural Tanzania [36], demonstrating its suitability in different contextual environments.

The facilitation methods used in the implementation process seemed to enhance the implementation and motivation of the OTs. These methods can be easily transferred to any implementation process in healthcare where innovative healthcare interventions are designed and implemented into practice. The model and principles of the intervention F@ce™ could provide specialized rehabilitation services for different diagnostic groups in both high- and low- income settings, especially in rural areas where patients have to travel long distances to the care providers. However, the interventions need to be carefully planned and adapted with regard to cultural differences worldwide. Because of the positive impression of the implementers and the confidence in the intervention’s potential to reduce inequality in access to healthcare services, the authors suggest that the SMS service is further evaluated when combined with more conventional methods. In order to get more comprehensive knowledge of the costs, the authors also suggest that an economic evaluation is conducted in order to make a fair comparison regarding rehabilitation measures in relation to conventional rehabilitation methods.

## Strengths and limitations

Using the combined theoretical framework and investigating all the constructs of i-PARIHS resulted in rich data and minimizing the risk of losing essential information on what may have influenced the implementation of the intervention. Another strength in the study was that the evaluators were not participants in the original implementing team, were blinded to the trial outcome and therefore reduced the possible bias. However, one limitation could be that the clients’ and caregivers’ perceptions were not included, and therefore this study provides only a partial picture of the intervention and its implementation.

## Conclusions

The intervention was partially delivered in accordance with the logic model for the project, where the implementation process was influenced by several barriers in the context, such as technical setbacks. However, there were also several mediators in the process, including strong facilitation and motivated participants, driving the project forward. For future research, expanding the feasibility study to a full-scale randomized controlled trial is suggested, but some adjustments are recommended, such as improved information dissemination to stakeholders and the use of a local internet server.

## Additional files

Additional file 1: The modelling process of the intervention A description of the process when the intervention was modelled from a Swedish client-centred ADL intervention into the Uganda context. (DOCX 15 kb)

Additional file 2: The logic model for developing the intervention. The Logic Model for developing the family based mobile phone supported rehabilitation intervention for persons with stroke in Uganda. (DOCX 16 kb)

## Abbreviations

ADL: Activities in daily living
ICT: Information- and communication technology
i-PARIHS: Integrated-Promoting Action on Research Implementation in Health Services
IT: Information technology
MRC: Medical Research Council
OT(s): Occupational therapist(s)
SDG(s): Sustainable Development Goal(s)
SMS: Short message service
