# Supplementary material for: What’s in it for me? A process evaluation of the implementation of a mobile phone-supported intervention after stroke in Uganda
Source: BMC Public Health. 2019 May 14;19:562. doi: 10.1186/s12889-019-6849-3 (PMC6518972; doi:10.1186/s12889-019-6849-3)
Supplement: Supplementary file 1 — The modelling process of the intervention A description of the process when the intervention was modelled from a Swedish client-centred ADL intervention into the Uganda context. (DOCX 15 kb) [file 12889_2019_6849_MOESM1_ESM.docx]

# Additional File 1. The modelling process of the intervention

| Step 1 | April 2015 | Modelling the intervention in Sweden based on two Swedish RCTs and a qualitative study on mobile use among persons with stroke in Uganda |
| --- | --- | --- |
| Step 2 | April 2015 | First version of the intervention modelled by four researchers |
| Step 3 | May 2015 | The project leader and the local facilitator had a meeting with the IT-specialists in Sweden confirming the intervention |
| Step 4 | September 2015 | Three days´ workshop in Uganda. Participants: three researchers from Sweden, one IT-specialist from Sweden and one from Uganda, one research coordinator from Uganda. Five OTs and one Ugandan professor participated one day modelling the intervention into the Ugandan context |
| Step 5 | 2016 | Two researchers conducted five several meetings with IT- specialists in Sweden. |
| Step 6 | February 2016 | Workshop in Uganda for five therapists. *One therapist withdraw himself after the first day. Another came in the second week, participated for five half days. SWOT-analysis was done. The SMS application wasn´t ready for testing. The Ugandan technician developed a protocol app for the researcher. |
| Step 7 | September 2016 | Two researchers from Sweden and one from Uganda did a refresh day of the intervention with one of the trained OTs and one OT who had administrative tasks. One OT couldn´t attend and one OT attended only part of the day. The content of the day was: 1. Updated on the ongoing project. 2. Presented the findings from the Swedish part of the research and two additional qualitative studies. 3. A new SWOT analysis was done and compared to the old one. Two OTs and the local facilitator was interviewed. |
