# Supplementary material for: What’s in it for me? A process evaluation of the implementation of a mobile phone-supported intervention after stroke in Uganda
Source: BMC Public Health. 2019 May 14;19:562. doi: 10.1186/s12889-019-6849-3 (PMC6518972; doi:10.1186/s12889-019-6849-3)
Supplement: Supplementary file 2 — The logic model for developing the intervention. The Logic Model for developing the family based mobile phone supported rehabilitation intervention for persons with stroke in Uganda. (DOCX 16 kb) [file 12889_2019_6849_MOESM2_ESM.docx]

# Additional File 2. The Logic Model for developing the family based mobile phone supported rehabilitation intervention for persons with stroke in Uganda.

| **Resources** | **Activities** | **Outputs** | **Short and Long term outcomes** | **Impact** |
| --- | --- | --- | --- | --- |
| - Skilled OTs & PTs - Funds available - ICT specialists - Clients and family - Enabling environment - MDT - Knowledge on use mobile phones - Mobile phones - Good literacy skills - Coordinator - Organizational plan - Knowledge of stroke - Scientific techniques for stroke rehabilitation | - Training workshops for OTS. - Support from researchers - Regular meetings - Assessments - Interviews - Home visits & follow ups - Setting goals with clients - Training clients & caregivers - Making feedback - Good record keeping - Evaluation of goals - Demonstration of techniques - Build therapeutic relationships and maintain it via telephone | - Monitoring of interventions - Training of therapist done - Support from researchers received - Regular meetings held - Assessments done - Interviews carried out - Home visits & follow ups carried out - Setting goals with clients done - Training clients & family members - Making feedback - Evaluation of goals | **Short term**   - Increased knowledge of care for clients and family - Enhanced therapist client relationships - Increased client compliance to rehab services   **Long term**   - Improved quality of life of persons with stroke - Accessible & sustainable rehab services - Increased therapist output - Reduced burden of stroke to person and family. - Increased independence & participation. | - Improved rehabilitation services - Provides a model for rehabilitation - Increased awareness about stroke rehabilitation. - Increased therapists motivation to work - Follow-up available for stroke parsons. - Increased number of stroke receiving rehabilitation - Increased satisfaction with rehabilitation of people with stroke - Encourage transfer of rehab in natural context of home environment as opposed to rehab facilities - Sustainable cost effective use of rehabilitation personnel - Families with stroke patients will have improved knowledge & tools to support themselves in everyday life. |
